# Fear of COVID-19 Scale—Associations of Its Scores with Health Literacy and Health-Related Behaviors among Medical Students

**DOI:** 10.3390/ijerph17114164

**Published:** 2020-06-11

**Authors:** Hiep T. Nguyen, Binh N. Do, Khue M. Pham, Giang B. Kim, Hoa T.B. Dam, Trung T. Nguyen, Thao T.P. Nguyen, Yen H. Nguyen, Kristine Sørensen, Andrew Pleasant, Tuyen Van Duong

**Affiliations:** 1 Faculty of Public Health, Pham Ngoc Thach University of Medicine, Ho Chi Minh 725-10, Vietnam; nguyenthanhhiep@pnt.edu.vn; 2 Pham Ngoc Thach Clinic, Pham Ngoc Thach University of Medicine, Ho Chi Minh 725-10, Vietnam; 3 President Office, Pham Ngoc Thach University of Medicine, Ho Chi Minh 725-10, Vietnam; 4 Department of Infectious Diseases, Vietnam Military Medical University, Hanoi 121-08, Vietnam; nhubinh.do@vmmu.edu.vn; 5 Division of Military Science, Military Hospital 103, Hanoi 121-08, Vietnam; 6 Faculty of Public Health, Hai Phong University of Medicine and Pharmacy, Hai Phong 042-12, Vietnam; pmkhue@hpmu.edu.vn; 7 President Office, Hai Phong University of Medicine and Pharmacy, Hai Phong 042-12, Vietnam; 8 Institute of Preventive Medicine and Public Health, Hanoi Medical University, Hanoi 115-20, Vietnam; kimbaogiang@hmu.edu.vn; 9 Center for Assessment and Quality Assurance, Hanoi Medical University, Hanoi 115-20, Vietnam; 10 Department of Psychiatry, Thai Nguyen University of Medicine and Pharmacy, Thai Nguyen 241-17, Vietnam; baohoaydtn@gmail.com; 11 School of Medicine and Pharmacy, Vietnam National University, Hanoi 113-09, Vietnam; thanhtrungnguyen.smp@gmail.com; 12 Health Management Training Institute, Hue University of Medicine and Pharmacy, Thua Thien Hue 491-20, Vietnam; ntpthao.hmti@huemed-univ.edu.vn; 13 Department of Health Economics, Corvinus University of Budapest, 1093 Budapest, Hungary; 14 Department of Pharmacology and Clinical Pharmacy, Can Tho University of Medicine and Pharmacy, Can Tho 941-17, Vietnam; nhyen@ctump.edu.vn; 15 Department of Pharmacy, Can Tho University of Medicine and Pharmacy Hospital, Can Tho 941-17, Vietnam; 16 Global Health Literacy Academy, Viengevej 100, 8240 Risskov, Denmark; contact@globalhealthliteracyacademy.org; 17 Health Literacy Media, St. Louis, MO 63101, USA; apleasant@healthliteracy.media; 18 School of Nutrition and Health Sciences, Taipei Medical University, Taipei 110-31, Taiwan

**Keywords:** fear of COVID-19, academic year, medical students, psychological health, mental health, health literacy, lifestyles, behaviors, principal component analysis, Vietnam

## Abstract

The coronavirus disease 2019 (COVID-19) pandemic causes fear, as its immediate consequences for the public have produced unprecedented challenges for the education and healthcare systems. We aimed to validate the fear of COVID-19 scale (FCoV-19S) and examine the association of its scores with health literacy and health-related behaviors among medical students. A cross-sectional study was conducted from 7 to 29 April 2020 on 5423 students at eight universities across Vietnam, including five universities in the North, one university in the Center, two universities in the South. An online survey questionnaire was used to collect data on participants’ characteristics, health literacy, fear of COVID-19 using the FCoV-19S, and health-related behaviors. The results showed that seven items of the FCoV-19S strongly loaded on one component, explained 62.15% of the variance, with good item–scale convergent validity and high internal consistency (Cronbach’s alpha = 0.90). Higher health literacy was associated with lower FCoV-19S scores (coefficient, B, −0.06; 95% confidence interval, 95%CI, −0.08, −0.04; *p* < 0.001). Older age or last academic years, being men, and being able to pay for medication were associated with lower FCoV-19S scores. Students with higher FCoV-19S scores more likely kept smoking (odds ratio, OR, 1.11; 95% CI, 1.08, 1.14; *p* < 0.001) or drinking alcohol (OR, 1.04; 95% CI, 1.02, 1.06; *p* < 0.001) at an unchanged or higher level during the pandemic, as compared to students with lower FCoV-19S scores. In conclusion, the FCoV-19S is valid and reliable in screening for fear of COVID-19. Health literacy was found to protect medical students from fear. Smoking and drinking appeared to have a negative impact on fear of COVID-19. Strategic public health approaches are required to reduce fear and promote healthy lifestyles during the pandemic.

## 1. Introduction

The coronavirus disease 2019 (COVID-19) pandemic has caused a huge burden to governments, organizations, and individuals [1,2,3]. According to the report of the World Health Organization on 3 May 2020, the total confirmed cases were 3,349,786, with 238,628 deaths; among these, Vietnam reported 270 cases [4,5]. The pandemic causes fear, panic, and mental health problems in the public [6,7,8] and healthcare workers [9,10].

During the pandemic, myths and misinformation largely concern the public [6,11,12]. This might affect public’s psychological health [6,11,12]. People with a higher degree of health literacy are likely to have a better perception of health information [13]. Health literacy has been shown as a protective factor of mental health (e.g., depression) in a previous study [14].

Medical students are future healthcare providers who need comprehensive capacities to improve their self-care and to strengthen patients’ autonomy, participation, and self-management abilities [15,16]. Public health measures such as quarantine and social distancing are implemented in all countries to contain the spread of COVID-19 [17,18,19]. However, these approaches have negative effects on people’s mental health [20]. The general public is urged to maintain social distancing, work, and study remotely if possible, during the pandemic. Healthcare professionals in the meantime still have to work and continue providing care. They are more likely to have psychological health problems [21,22,23,24].

Amid the COVID-19 pandemic, all affected countries have mobilized and reallocated resources to respond to the COVID-19 emergency [25,26,27]. Rearrangement of healthcare personnel and changes in how medical students are encouraged which have occurred as part of the pandemic response [25,26,28,29]. In addition, students who are taking clinical practicum courses might be at a higher risk of infections. Moreover, medical students’ mental health has already been demonstrated to a problem worldwide, including in Vietnam, even prior to the pandemic [30].

Fear of COVID-19 causes healthcare access delays [8] or even suicide [31]. To mitigate and contain COVID-19 spread and its unfavorable mental health consequences, it is essential to detect adverse psychological problems (e.g., fear) and implement appropriate interventions at an early stage of their occurrence [32,33]. A tool was developed to screen for fear of COVID-19 [34]. However, this tool has not been validated for use in Vietnam or other countries. Therefore, we aimed to validate the fear of COVID-19 scale (FCoV-19S) and examine, using an online survey, the association of its scores with health literacy and health-related behaviors among medical students at eight universities across Vietnam.

## 2. Methods

### 2.1. Study Design and Settings

A cross-sectional study was conducted on medical students from 7 to 29 April 2020 using online-based survey questionnaires. Students were recruited from eight universities across Vietnam, including five universities in the North, one university in the Center, and two universities in the South.

### 2.2. Study Participants

**Figure 1:**
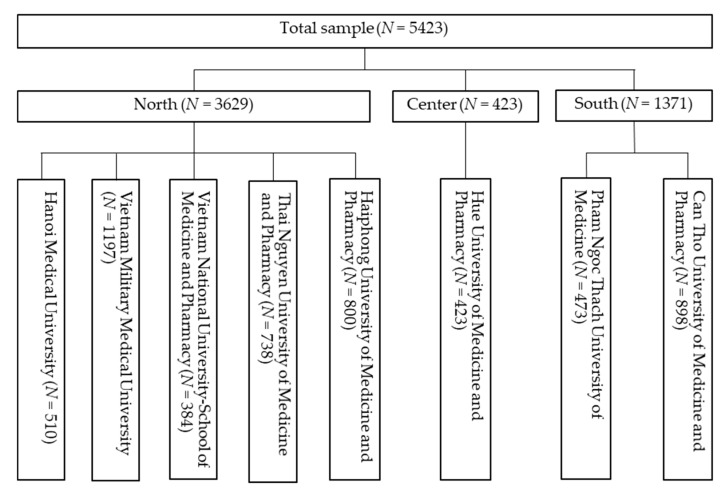
Flow chart of study sampling.

A total of 5423 medical students participated in the survey, including 510 (15.1%) from Hanoi Medical University, 1197 (39.5%) from Vietnam Military Medical University, 384 (86.5%) from Vietnam National University- School of Medicine and Pharmacy, in Hanoi city; 738 (26.1%) from Thai Nguyen University of Medicine and Pharmacy, in Thai Nguyen Province; 800 (25.4%) from Haiphong University of Medicine and Pharmacy, in Haiphong city; 423 (11.1%) from Hue University of Medicine and Pharmacy, in Thua Thien Hue province; 473 (9.6%) from Pham Ngoc Thach University of Medicine, in Ho Chi Minh city; 898 (13.7%) from Can Tho University of Medicine and Pharmacy, in Can Tho city. The recruitment of the study sample is elucidated in Figure 1.

### 2.3. Assessments and Measurements

#### 2.3.1. Social Demographics and Clinical Indicators

Students were asked about their age (years), gender (women vs. men), body height (cm), weight (kg). Body mass index (BMI, kg/m^2^) was calculated. They were also asked about their academic year (from year 1 to year 6) and suspected levels of exposure to COVID-19, evaluated as F1 (exposed to a person with confirmed COVID-19), F2 (exposed to F1), F3 (exposed to F2), F4 (exposed to F3), F5 (exposed to F4), to F6 (not suspected), according to the definition of the Ministry of Health in Vietnam [5]. Students were also asked if the experienced suspected COVID-19 symptoms (S-COVID-19-S) [35], including common symptoms (fever, cough, dyspnea) and less common symptoms (myalgia, fatigue, sputum production, confusion, headache, sore throat, rhinorrhea, chest pain, hemoptysis, diarrhea, and nausea/vomiting). If students had any of those symptoms, they were classified as having S-COVID-19-S. The Charlson comorbidity index was used to assess comorbidity [36,37].

#### 2.3.2. Health-Related Behaviors

Students were asked about their current health-related behaviors as compared with those before the pandemic, including tobacco smoking (never/stop/less vs. unchanged or more), alcohol drinking (never/stop/less vs. unchanged or more), physical activity level (never/stop/less vs. unchanged or more), and eating behavior (less healthy vs. unchanged or healthier).

#### 2.3.3. Health Literacy

Health literacy (HL) was assessed using the short-form HL questionnaire (HLS-SF12) consisting of 12 items. The HLS-SF12 was used as a comprehensive measure of HL for the evaluation of students’ ability to access, understand, appraise, and apply health information on healthcare, disease prevention, and health promotion [38]; it is in agreement with the original framework of comprehensive HL [39]. In addition, the tool was validated and used in Asian countries [38,40], including in Vietnam [14,41,42]. Students responded to each item on 4-point Likert scales from 1 = very difficult to 4 = very easy. The HL index score was standardized to a metric from 0 to 50, using formula (1), with a higher score indicating better HL [43]:

*Index* = (*Mean* − *1*) × (*50*/*3*) (1)

where *Index* is the specific index calculated, *Mean* is the mean of all participating items for each individual, *1* is the minimal possible value of the mean (leading to a minimum value of the index of 0), *3* is the range of the mean, and *50* is the chosen maximum value of the new metric.

#### 2.3.4. Fear of COVID-19

The fear of COVID-19 as an immediate consequence of the pandemic was assessed using the FCoV-19S, which is reliable and valid in assessing COVID-19 fear among the general population [34]. The questionnaire was translated into Vietnamese by researchers. The content was then validated by an expert panel (one psychiatrist, 10 medical doctors, 7 nurses, and 5 public health professionals). The expert panel suggested keeping the original rating scale and scoring. The tool consists of 7 items evaluated on a 5-point Likert scale, with 1 = “strongly disagree”, 2 = “disagree”, 3 = “neither disagree or agree”, 4 = “agree”, 5 = “strongly agree”. The total score is the sum of the scores of the 7 items, ranging from 7 to 35, with a higher score indicating greater fear of COVID-19.

In addition, anxiety disorder was assessed using the Generalized Anxiety Disorder scale with seven items (GAD-7) [44]. The tool was validated and used in Vietnam [45]. Students were asked about how often (during the last 2 weeks) they experienced seven symptoms based on a 4-point Likert scale, with 0 = “not at all”, 1 = “several days”, 2 = “more than half the days”, 3 = “nearly every day”. The total GAD-7 score ranges from 0 to 21. Students were classified as having anxiety disorder if they had a GAD-7 score ≥8 [46,47].

### 2.4. Data Collection Procedure

We purposely selected 5 public universities in the North, 1 public university in the Center, and 2 public universities in the South of Vietnam. Medical students were invited and encouraged to participate in the survey by lecturers of the university. Researchers (lecturers) at each university sent the online survey link to the contact students of each class via email, Messenger, or Zalo. The link was then sent to other students. The students voluntarily took the survey. It took about 5 min to complete the survey questionnaires. All questions on the online survey were mandatory; therefore, there are no missing data in our study. The collected data were coded, cleaned, and analyzed by researchers confidentially.

### 2.5. Data Analysis

Firstly, the distribution of the studied variables was explored using descriptive analysis. Mean, standard deviation, or percentages were reported appropriately.

*Construct validity:* To examine the construct of the FCoV-19 scale, principal component analysis (PCA) was used. The Kaiser–Meyer–Olkin Measure of Sampling Adequacy (KMO) was used to determine the suitability of the data for component analyses and was set to be greater than 0.60, while the Bartlett’s Test of Sphericity value was set to be less than 0.05 [48]. One component representing the unidimensional scale of FCoV-19S was retained, while the oblique rotation (Promax) method was used. Factor loadings of seven items of the FCoV-19 scale and percentage of variance were reported.

*Convergent validity:* The correlation between the FCoV-19 scale’s scores and its seven items was determined by the Spearman’s correlation coefficient.

*Discriminant validity:* Anxiety disorder was used to evaluate the discriminant validity of the FCoV-19S. A receiver operating characteristic (ROC) curve analysis was run with anxiety (GAD ≥ 8) as the reference to estimate the area under the ROC curve (AUC).

*Reliability analysis:* The Cronbach’s alpha test was used to assess the internal consistency, with satisfactory reliability corresponding to a value ≥0.70 [49].

*Floor and ceiling effects of the FCoV-19 scale:* The percentages of respondents who scored the lowest score or the highest score were calculated. The minimal floor and ceiling effects (<15%) were recommended [50].

*Associations between FCoV-19 and other factors:* One-way ANOVA test was used to compare the distribution of FCoV-19S scores between categories of the studied variables. In addition, simple and multiple linear regression models were utilized to examine the predictors of fear of COVID-19. The factors that were included in the multiple regression analysis were those that showed association with FCoV-19S scores at *p* < 0.20 in the simple regression model [51]. The regression coefficient (B) and 95% confidence interval (95%CI) were reported. Furthermore, the associations between FCoV-19S scores and health-health related behaviors were examined using simple and multiple logistic regression models. The odds ratio (OR) and 95%CI were reported.

Data were analyzed using the IBM SPSS Version 20.0 (IBM Corp, Armonk, NY, USA). The significance level was set at *p* < 0.05.

### 2.6. Ethical Consideration

The study protocol was approved by the Institutional Ethical Review Committee of Hanoi School of Public Health, Vietnam (IRB No. 133/2020/YTCC-HD3). The consent form was obtained from the students before their participation.

## 3. Results

### 3.1. Participants’ Characteristics

The average age of the students was 22.0 ± 2.0. Of all, 47.9% were men; for 53.9% of the students it was very or fairly easy to pay for their necessary medications. The prevalence of underweight and overweight/obese was 17.4% and 8.2%, respectively. In addition, 18.9% of the students presented with suspected COVID-19 symptoms. The proportions of students eating, smoking, drinking, or exercising at an ‘unchanged or higher level were 93.0%, 3.1%, 6.95%, or 68.1%, respectively (Table 1).

### 3.2. Psychometric Properties of FCoV-19

The KMO value for the whole scale was 0.86, while, for individual items, the values ranged from 0.77 to 0.94 and were above the acceptable limit of 0.50. The Bartlett’s Test of Sphericity value was less than 0.001, at a satisfactory level.

Seven items were loaded on one component with the percentage of variance of 62.15%. The factor loadings of seven items are shown in Table 2. The average communality value of 0.62 was at an adequate level, demonstrating the accuracy of the approach [52].

**Figure 2:**
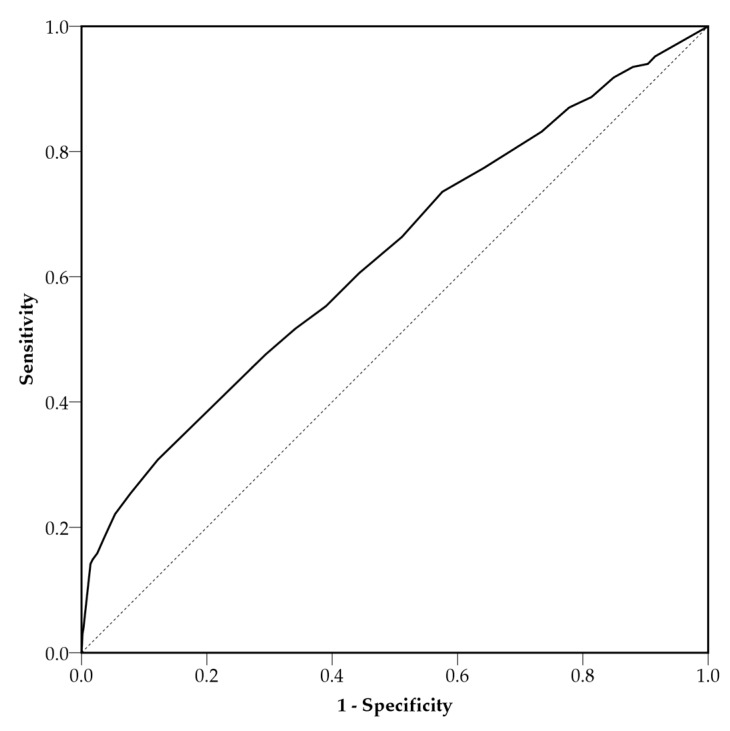
The receiver operating characteristic curve of the FCoV-19S predicting anxiety (GAD ≥ 8). The area under the curve is 0.63.

The correlations between each item and the scale ranged from 0.66 to 0.84. In addition, the AUC was 0.63 (0.60–0.66), thus larger than 0.50 (Table 2; Figure 2). This indicates satisfactory discrimination validity of the FCoV-19S [53].

The reliability was at a high level, with Cronbach’s alpha value of 0.90. There were no significant floor or ceiling effects, with 8.20% of the participants at the lowest potential response, and 0.40% at the highest potential response, which were less than 15% (Table 2).

### 3.3. Factors Associated with FCoV-19

The score of fear of COVID-19 significantly varied by categories of age, gender, ability to pay for medication, BMI, academic year, smoking, drinking, and anxiety (Table 1). The results in Table 3 show that older age (23–26 years), being men, great or fair easiness to pay for medication, later academic years, higher health literacy score were significantly negatively associated with FCoV-19S scores, whereas smoking or drinking at an unchanged or higher level was positively associated with the FCoV-19S scores in the bivariate model. To avoid multicollinearity, correlation among the covariates was checked. A strong correlation between age and academic year (rho = 0.84) was found (Table S1). We selected age in the multivariate analysis together with other covariates that were associated with FCoV-19S at *p* < 0.20. The results showed that age of 23–26 years (coefficient, B, −0.96; 95% confidence interval, 95%CI, −1.24, −0.67; *p* < 0.001), being men (B, −0.68; 95%CI, −0.97, −0.38; *p* < 0.001), great or fair easiness to pay for medication (B, −0.45; 95%CI, −0.73, −0.17; *p* < 0.002), higher health literacy (B, −0.06; 95%CI, −0.08, −0.04; *p* < 0.001; Table 3) had lower scores on the FCoV−19S as compared to their respective counterparts. The adjusted R-square was 0.022.

Finally, the students with higher FCoV-19S scores were more likely to be smoking at an unchanged or higher level (odds ratio, OR, 1.11; 95% CI, 1.08, 1.14; *p* < 0.001) and drinking at an unchanged or higher level (OR, 1.04; 95% CI, 1.02, 1.06; *p* < 0.001; Table 4). In other words, a 1-point increment of the FCoV-19S score was associated with an 11% or 4% greater likelihood of smoking or drinking at the same or higher level, respectively. In addition, results of the Hosmer and Lemeshow test of the goodness of fit in multiple logistic regression models showed *p*-values of 0.64 (for eating behavior), 0.07 (for smoking behavior), 0.47 (for drinking behavior), and 0.11 (for exercise), which were all larger than 0.05. This indicates that the models well fit the data [53].

## 4. Discussion

The results of our study show that the fear of COVID-19 scale is a unidimensional, valid, and reliable survey tool to assess the fear of students during the COVID-19 pandemic. The tool demonstrates higher values of factor loadings, item–scale convergent validity, internal consistency, and better floor and ceiling effects in the current study than that in a previous study [34].

Students at older age and later academic year had lower scores of fear than those at younger age and earlier academic year. This could be explained by the fact that senior students had a better knowledge about the disease, medical skills, and prevention measures than junior ones, which could further protect them from fear of COVID-19. In addition, male students reported a lower score of fear as compared with female students. This might be explained by the fact that women usually sustain a greater burden than men during a pandemic, including house works, caregiving role, or domestic violence [54]. In addition, women usually suffer from more stressful life events than men [55,56]. Previous studies illustrated that the prevalence of anxiety and depression of graduate female students was higher than that of male students [57,58]. It suggests that universities should have a strategic approach to protect the mental health of students, with additional focus on that of female students [57,59].

In the current study, students with a greater ability to pay for necessary medication and healthcare services had a lower score of fear. This agrees with a previous study which demonstrated that college students with family income stability had a lower likelihood of psychological problems during the COVID-19 pandemic [60].

The results of the current study show that students with a higher score of fear are more likely to smoke or drink. The finding is in line with a previous study reporting that mental disorders were a risk factor for substance use, abuse, and dependence [61]. In addition, a previous study of 41 low–middle-income countries showed that people with a higher stress level had a higher likelihood of smoking [62]. Furthermore, cigarette smoking was found to help to ease negative emotions in unpleasant events [62,63]. Additionally, alcohol drinking can temporarily help to reduce tension, and stress [64]. Importantly, lifestyle factors demonstrated a significant association with the clinical course of COVID-19 [65,66]. Therefore, strategic public health interventions are needed to reduce fear and reduce substance use or abuse among medical students, which might further protect their health.

Finally, health literacy showed a protective effect on fear in our study. Health literacy has been recognized as a critical skill in evaluating online health information [67], especially in our digital world characterized by diverse information and sources [68]. Besides, higher health literacy was associated with a better health status [69,70], reduced health inequities, and improved health and well-being [71,72]. Furthermore, a previous study showed that health literacy can help to protect people’s mental health and improve quality of life during the COVID-19 pandemic [14]. Therefore, enhancing medical students’ health literacy skills is considered a strategic approach to reducing fear and improving their health and wellbeing. It is important for people, especially healthcare workers, to have adequate possibility to access, analyze, and apply health information during the COVID-19 pandemic to protect their own health and that of people they take care of. Therefore, health literacy should be seen as a key element of social responsibility and solidity and an essential tool for both information receivers and providers in order to mitigate and contain the current pandemic and potential future ones [73]. An interdisciplinary approach to improving health literacy is more important before than during a pandemic [74].

The current study has some limitations. Firstly, the survey was conducted online, and we could not diagnose any psychological disorder in the students (e.g., anxiety or depression), which would have help to check the sensitivity and specificity of the FCoV-19 scale. Secondly, causality cannot be drawn from this study, which used a cross-sectional design. Thirdly, test–retest reliability could not be assessed because of the nature of the study design. This is similar to a previous study conducted in China with limited resources and the urgent need of the evidence during COVID-19 pandemic [75]. We surveyed a large sample of students at eight universities across Vietnam, but the study sample was not randomly selected. Therefore, the finding should be generalized to medical students with caution. Furthermore, the low R-square value (0.02 or 2%) and the relatively low AUC value (0.63) may indicate that the predictive capability of these models is limited and other variables not included in this study may increase it. Future studies are required to address the above-mentioned limitations. Despite these limitations, our findings help describe the phenomenon of the current pandemic, raise hypotheses for further research related to fear and lifestyle changes during pandemics, and suggest means to increase preparedness for possible future pandemics.

## 5. Conclusions

The current study shows that the fear of COVID-19 scale is a valid and reliable tool to screen for fear among medical students in Vietnam. Factors such as older age or later academic year, being male, a greater ability to pay for medication, and a higher degree of health literacy may protect medical students from fear during the pandemic. In addition, students with a higher score of fear more likely had unhealthy lifestyles, such as smoking and drinking alcohol. Strategic public health interventions are suggested to reduce fear and promote healthy lifestyles which may further protect students’ health and wellbeing.

## Figures and Tables

**Table 1:** Participants’ characteristics and scores on the fear of COVID-19 scale (FCoV-19S).

Variables	Total (*n* = 5423)	FCoV-19S (*n* = 5423)	
	Frequency (%)	Mean ± SD	*p* *
Age, year			<0.001
19–22	3162 (58.3)	17.0 ± 5.3	
23–26	2261 (41.6)	16.0 ± 5.1	
Gender			<0.001
Women	2821 (52.0)	17.0 ± 4.8	
Men	2602 (47.9)	16.2 ± 5.6	
Ability to pay for medication			<0.001
Very or fairly difficult	2496 (46.0)	16.9 ± 5.3	
Very or fairly easy	2927 (53.9)	16.4 ± 5.1	
BMI, kg/m^2^			0.018
Underweight (BMI < 18.5)	945 (17.4)	17.0 ± 4.8	
Normal weight (18.5 ≤ BMI < 25.0)	4034 (74.3)	16.5 ± 5.3	
Overweight/obese (BMI ≥ 25.0)	444 (8.2)	16.7 ± 5.5	
Academic year			<0.001
1	1171 (21.5)	17.0 ± 5.3	
2	773 (14.2)	17.3 ± 5.5	
3	762 (14.0)	17.2 ± 5.2	
4	738 (13.6)	16.9 ± 5.2	
5	920 (16.9)	15.9 ± 5.2	
6	1059 (19.5)	15.8 ± 4.9	
S-COVID-19-S **			0.186
No	4396 (81.0)	16.6 ± 5.3	
Yes	1027 (18.9)	16.8 ± 5.0	
Comorbidity			0.768
None	5180 (95.5)	16.6 ± 5.2	
One or more	243 (4.5)	16.5 ± 5.2	
Eating behavior ***			0.205
Eat less healthy	377 (7.0)	16.9 ± 5.4	
Unchanged or healthier	5046 (93.0)	16.6 ± 5.2	
Smoking ***			<0.001
Never, stopped, or smoke less	5255 (96.9)	16.5 ± 5.1	
Unchanged or smoke more	168 (3.1)	19.4 ± 7.6	
Drinking alcohol ***			0.001
Never, stopped, or drink less	5048 (93.0)	16.5 ± 5.1	
Unchanged or drink more	375 (6.9)	17.5 ± 6.7	
Physical activity ***			0.599
Never, stopped, or exercise less	1728 (31.8)	16.6 ± 5.4	
Unchanged or exercise more	3695 (68.1)	16.6 ± 5.1	
Anxiety			<0.001
GAD < 8	5007 (92.3)	16.4 ± 5.1	
GAD ≥ 8	416 (7.7)	19.3 ± 6.3	
FCoV-19, mean ± SD	16.7 ± 5.3		
HL index, mean ± SD	34.7 ± 7.0		

Abbreviations: BMI, body mass index; COVID-19, coronavirus disease 2019; S-COVID-19-S, suspected coronavirus disease 2019 symptoms; FCoV-19S, fear of coronavirus disease 2019 scale; GAD, generalized anxiety disorder; SD, standard deviation; HL, health literacy. * Result of One-Way ANOVA test. ** Suspected COVID-19 symptoms including common symptoms (fever, cough, dyspnea), less common symptoms (myalgia, fatigue, sputum production, confusion, headache, sore throat, rhinorrhea, chest pain, hemoptysis, diarrhea, and nausea/vomiting). *** People were asked whether their lifestyles were worse, better, or unchanged during the COVID-19 pandemic as compared to those before the pandemic.

**Table 2:** Construct and convergent validity, internal consistency, and floor and ceiling effects of the FCoV-19 scale (*n* = 5423).

Items	FCoV-19 Scale
1. I am most afraid of COVID-19.	0.84
2. It makes me uncomfortable to think about COVID-19.	0.83
3. My hands become clammy when I think about COVID-19.	0.81
4. I am afraid of losing my life because of COVID-19.	0.81
5. When watching news and stories about COVID-19 on social media, I become nervous or anxious.	0.78
6. I cannot sleep because I’m worrying about getting COVID-19.	0.76
7. My heart races or palpitates when I think about getting COVID-19.	0.69
Percentage of variance, %	62.15
Item–scale convergent validity, mean of Rho (range)	0.77 (0.66–0.84)
AUC (95%CI), GAD ≥ 8 as the reference	0.63 (0.60–0.66)
Internal consistency, Cronbach’s alpha	0.90
Floor effects, %	8.20
Ceiling effect, %	0.40

Abbreviations: Rho, Spearman’s correlation coefficient; AUC, area under the receiver operating characteristic curve; CI, confidence interval.

**Table 3:** Predictors of the fear of COVID-19 (*n* = 5423).

Variables	FCoV-19S			
	Simple Model		Multiple Model	
	B (95%CI)	p *	B (95%CI)	p *
Age, year				
19–22	0.00		0.00	
23–26	−1.02 (−1.31, −0.74)	<0.001	−0.96 (−1.24, −0.67)	<0.001
Gender				
Women	0.00			
Men	−0.71 (−0.99, −0.43)	<0.001	−0.68 (−0.97, −0.38)	<0.001
Ability to pay for medication				
Very or fairly difficult	0.00			
Very or fairly easy	−0.55 (−0.83, −0.26)	<0.001	−0.45 (−0.73, −0.17)	0.002
BMI, kg/m^2^				
Underweight (BMI < 18.5)	0.54 (0.16, 0.91)	0.005	0.29 (−0.09, 0.67)	0.133
Normal weight (18.5 ≤ BMI < 25.0)	0.00			
Overweight/obese (BMI ≥ 25.0)	0.21 (−0.31, 0.73)	0.421	0.48 (−0.04, 1.00)	0.069
Academic year				
1	0.00			
2	0.31 (−0.17, 0.79)	0.203		
3	0.23 (−0.25, 0.71)	0.349		
4	−0.02 (−0.50, 0.46)	0.933		
5	−1.10 (−1.56, −0.65)	<0.001		
6	−1.18 (−1.62, −0.74)	<0.001		
S-COVID-19-S **				
No	0.00			
Yes	0.24 (−0.12, 0.6)	0.186	0.07 (−0.29, 0.43)	0.708
Comorbidity				
None	0.00			
One or more	−0.1 (−0.78, 0.58)	0.768		
HL index, 1-score increment	−0.07 (−0.09, −0.05)	<0.001	−0.06 (−0.08, −0.04)	<0.001

Abbreviations: B, regression coefficient; * Results of simple and multiple linear regression models. ** Suspected COVID-19 symptoms including common symptoms (fever, cough, dyspnea), less common symptoms (myalgia, fatigue, sputum production, confusion, headache, sore throat, rhinorrhea, chest pain, hemoptysis, diarrhea, and nausea/vomiting).

**Table 4:** Association between fear of COVID-19 and lifestyles analyzed via logistic regression models (*n* = 5423).

FCoV-19S *	Unchanged or Eat Healthier **		Unchanged or Smoke More **		Unchanged or Drinking More **		Unchanged or Exercise More **	
	OR (95%CI)	*p*	OR (95%CI)	*p*	OR (95%CI)	*p*	OR (95%CI)	*p*
Model 1	0.99 (0.97, 1.01)	0.205	1.11 (1.08, 1.14)	<0.001	1.03 (1.01, 1.06)	0.001	1 (0.99, 1.01)	0.599
Model 2	0.99 (0.97, 1.01)	0.211	1.11 (1.08, 1.14)	<0.001	1.04 (1.02, 1.06)	<0.001	1 (0.99, 1.01)	0.608

Abbreviations: OR, odds ratio. * The association between FCoV-19S scores and lifestyle indicators by 1-point increments. ** The reference groups are ‘Eat less healthily’, ‘Never, stopped, or smoke less’, ‘Never, stopped, or drink less’, ‘Never, stopped, or exercise less’, appropriately. Model 1: Association between FCoV-19S scores and lifestyles. Model 2: Adjusted for age, gender, ability to pay for medication, and health literacy.

## Supplementary Materials

The following are available online at https://www.mdpi.com/1660-4601/17/11/4164/s1, Table S1: Spearman correlations among covariates (*N* = 5423).

## References

[1] The Lancet Infectious Diseases (2020). COVID-19: Endgames. Lancet Infect. Dis..

[2] Duan H., Wang S., Yang C. (2020). Coronavirus: Limit short-term economic damage. Nature.

[3] Rosenbaum L. (2020). The Untold Toll—The Pandemic’s Effects on Patients without Covid-19. N. Engl. J. Med..

[4] World Health Organisation (2020). Coronavirus Disease (COVID-2019) Situation Reports.

[5] Ministry of Health Coronavirus Disease (COVID-19) Outbreak in VIETNAM. https://ncov.moh.gov.vn/.

[6] Bao Y., Sun Y., Meng S., Shi J., Lu L. (2020). 2019-nCoV Epidemic: Address Mental Health Care to Empower Society. Lancet.

[7] Xu Z., Li S., Tian S., Li H., Kong L.-Q. (2020). Full spectrum of COVID-19 severity still being depicted. Lancet.

[8] Lazzerini M., Barbi E., Apicella A., Marchetti F., Cardinale F., Trobia G. (2020). Delayed access or provision of care in Italy resulting from fear of COVID-19. Lancet Child Adolesc. Health.

[9] Lai J., Ma S., Wang Y., Cai Z., Hu J., Wei N., Wu J., Du H., Chen T., Li R. (2020). Factors Associated With Mental Health Outcomes Among Health Care Workers Exposed to Coronavirus Disease 2019. JAMA Netw. Open.

[10] Pereira-Sanchez V., Adiukwu F., El Hayek S., Bytyçi D.G., Gonzalez-Diaz J.M., Kundadak G.K., Larnaout A., Nofal M., Orsolini L., Ramalho R. (2020). COVID-19 effect on mental health: Patients and workforce. Lancet Psychiatry.

[11] Shimizu K. (2020). 2019-nCoV, fake news, and racism. Lancet.

[12] Brooks S.K., Webster R.K., Smith L.E., Woodland L., Wessely S., Greenberg N., Rubin G.J. (2020). The psychological impact of quarantine and how to reduce it: Rapid review of the evidence. Lancet.

[13] Paige S.R., Krieger J.L., Stellefson M.L. (2017). The Influence of eHealth Literacy on Perceived Trust in Online Health Communication Channels and Sources. J. Health Commun..

[14] Nguyen H.C., Nguyen M.H., Do B.N., Tran C.Q., Nguyen T.T.P., Pham K.M., Pham L.V., Tran K.V., Duong T.T., Tran T.V. (2020). People with Suspected COVID-19 Symptoms Were More Likely Depressed and Had Lower Health-Related Quality of Life: The Potential Benefit of Health Literacy. J. Clin. Med..

[15] Kaper M.S., Reijneveld S.A., van Es F.D., de Zeeuw J., Almansa J., Koot J.A.R., de Winter A.F. (2019). Effectiveness of a Comprehensive Health Literacy Consultation Skills Training for Undergraduate Medical Students: A Randomized Controlled Trial. Int. J. Environ. Res. Public Health.

[16] Ali N.K., Ferguson R.P., Mitha S., Hanlon A. (2014). Do medical trainees feel confident communicating with low health literacy patients?. J. Community Hosp. Intern. Med. Perspect..

[17] Singh A., Shaikh A., Singh R., Singh A.K. (2020). COVID-19: From bench to bed side. Diabetes Metab. Syndr..

[18] Cheng H.-Y., Jian S.-W., Liu D.-P., Ng T.-C., Huang W.-T., Lin H.-H. (2020). For the Taiwan COVID-19 Outbreak Investigation Team. Contact Tracing Assessment of COVID-19 Transmission Dynamics in Taiwan and Risk at Different Exposure Periods Before and After Symptom Onset. JAMA Intern. Med..

[19] Lai S., Ruktanonchai N.W., Zhou L., Prosper O., Luo W., Floyd J.R., Wesolowski A., Santillana M., Zhang C., Du X. (2020). Effect of non-pharmaceutical interventions to contain COVID-19 in China. Nature.

[20] Armitage R., Nellums L.B. (2020). COVID-19 and the consequences of isolating the elderly. Lancet Public Health.

[21] Lima C.K.T., Carvalho P.M.M., Lima I., Nunes J., Saraiva J.S., de Souza R.I., da Silva C.G.L., Neto M.L.R. (2020). The emotional impact of Coronavirus 2019-nCoV (new Coronavirus disease). Psychiatry Res..

[22] Li Z., Ge J., Yang M., Feng J., Qiao M., Jiang R., Bi J., Zhan G., Xu X., Wang L. (2020). Vicarious traumatization in the general public, members, and non-members of medical teams aiding in COVID-19 control. Brain Behav. Immun..

[23] Wu W., Zhang Y., Wang P., Zhang L., Wang G., Lei G., Xiao Q., Cao X., Bian Y., Xie S. (2020). Psychological stress of medical staffs during outbreak of COVID-19 and adjustment strategy. J. Med. Virol..

[24] Pfefferbaum B., North C.S. (2020). Mental Health and the Covid-19 Pandemic. N. Engl. J. Med..

[25] Gallagher T.H., Schleyer A.M. (2020). “We Signed Up for This!”—Student and Trainee Responses to the Covid-19 Pandemic. N. Engl. J. Med..

[26] Bauchner H., Sharfstein J. (2020). A Bold Response to the COVID-19 Pandemic: Medical Students, National Service, and Public Health. JAMA.

[27] Fisher D., Wilder-Smith A. (2020). The global community needs to swiftly ramp up the response to contain COVID-19. Lancet.

[28] Baker D.M., Bhatia S., Brown S., Cambridge W., Kamarajah S.K., McLean K.A., Brindl N., Lapolla P., Pérez-Ajates S., Raubenheimer K. (2020). Medical student involvement in the COVID-19 response. Lancet.

[29] Rasmussen S., Sperling P., Poulsen M.S., Emmersen J., Andersen S. (2020). Medical students for health-care staff shortages during the COVID-19 pandemic. Lancet.

[30] Pham T., Bui L., Nguyen A., Nguyen B., Tran P., Vu P., Dang L. (2019). The prevalence of depression and associated risk factors among medical students: An untold story in Vietnam. PLoS ONE.

[31] Goyal K., Chauhan P., Chhikara K., Gupta P., Singh M.P. (2020). Fear of COVID 2019: First suicidal case in India!. Asian J. Psychiatr..

[32] Xiang Y.-T., Yang Y., Li W., Zhang L., Zhang Q., Cheung T., Ng C.H. (2020). Timely mental health care for the 2019 novel coronavirus outbreak is urgently needed. Lancet Psychiatry.

[33] Duan L., Zhu G. (2020). Psychological interventions for people affected by the COVID-19 epidemic. Lancet Psychiatry.

[34] Ahorsu D.K., Lin C.Y., Imani V., Saffari M., Griffiths M.D., Pakpour A.H. (2020). The Fear of COVID-19 Scale: Development and Initial Validation. Int. J. Ment. Health Addict..

[35] Editorial Team Overview of Novel Coronavirus (2019-nCoV). BMJ Best Practice. https://bestpractice.bmj.com/topics/en-gb/3000165.

[36] Quan H., Li B., Couris C.M., Fushimi K., Graham P., Hider P., Januel J.-M., Sundararajan V. (2011). Updating and Validating the Charlson Comorbidity Index and Score for Risk Adjustment in Hospital Discharge Abstracts Using Data From 6 Countries. Am. J. Epidemiol..

[37] Charlson M.E., Pompei P., Ales K.L., MacKenzie C.R. (1987). A new method of classifying prognostic comorbidity in longitudinal studies: Development and validation. J. Chronic Dis..

[38] Duong T.V., Aringazina A., Baisunova G., Nurjanah N., Pham T.V., Pham K.M., Truong T.Q., Nguyen K.T., Oo W.M., Su T.T. (2019). Development and validation of a new short-form health literacy instrument (HLS-SF12) for the general public in six Asian countries. Health Lit. Res. Pract..

[39] Sørensen K., Van den Broucke S., Brand H., Fullam J., Doyle G., Pelikan J., Slonszka Z. (2012). Health literacy and public health: A systematic review and integration of definitions and models. BMC Public Health.

[40] Duong T.V., Chang P.W., Yang S.-H., Chen M.-C., Chao W.-T., Chen T., Chiao P., Huang H.-L. (2017). A new comprehensive short-form health literacy survey tool for patients in general. Asian Nurs. Res. (Korean Soc. Nurs. Sci.).

[41] Duong T.V., Nguyen T.T.P., Pham K.M., Nguyen K.T., Giap M.H., Tran T.D.X., Nguyen C.X., Yang S.-H., Su C.-T. (2019). Validation of the Short-Form Health Literacy Questionnaire (HLS-SF12) and Its Determinants among People Living in Rural Areas in Vietnam. Int. J. Environ. Res. Public Health.

[42] Ho H.V., Hoang G.T., Pham V.T., Duong T.V., Pham K.M. (2020). Factors associated with health literacy among the elderly people in Vietnam. Biomed. Res. Int..

[43] HLS-EU Consortium Comparative Report of Health literacy in Eight EU Member States. The European Health Literacy Project 2009–2012. Maastricht University. https://www.healthliteracyeurope.net/hls-eu.

[44] Spitzer R.L., Kroenke K., Williams J.B., Löwe B. (2006). A brief measure for assessing generalized anxiety disorder: The GAD-7. Arch. Intern. Med..

[45] Pollack A.A., Weiss B., Trung L.T. (2016). Mental health, life functioning and risk factors among people exposed to frequent natural disasters and chronic poverty in Vietnam. BJPsych Open.

[46] Kroenke K., Spitzer R.L., Williams J.B., Monahan P.O., Löwe B. (2007). Anxiety disorders in primary care: Prevalence, impairment, comorbidity, and detection. Ann. Intern. Med..

[47] Plummer F., Manea L., Trepel D., McMillan D. (2016). Screening for anxiety disorders with the GAD-7 and GAD-2: A systematic review and diagnostic metaanalysis. Gen. Hosp. Psychiatry.

[48] Kaiser H.F. (1974). An index of factorial simplicity. Psychometrika.

[49] Cronbach L.J., Shavelson R.J. (2004). My current thoughts on coefficient alpha and successor procedures. Educ. Psychol. Meas..

[50] Terwee C.B., Bot S.D.M., de Boer M.R., van der Windt D.A.W.M., Knol D.L., Dekker J., Bouter L.M., de Vet H.C.W. (2007). Quality criteria were proposed for measurement properties of health status questionnaires. J. Clin. Epidemiol..

[51] Maldonado G., Greenland S. (1993). Simulation Study of Confounder-Selection Strategies. Am. J. Epidemiol..

[52] Field A. (2013). Discovering Statistics Using IBM SPSS Statistics.

[53] Hosmer D.W., Lemeshow S., Sturdivant R.X. (2013). Applied Logistic Regression.

[54] The L. (2020). The gendered dimensions of COVID-19. Lancet.

[55] Harkness K.L., Alavi N., Monroe S.M., Slavich G.M., Gotlib I.H., Bagby R.M. (2010). Gender differences in life events prior to onset of major depressive disorder: The moderating effect of age. J. Abnorm. Psychol..

[56] Conklin A.I., Guo S.X., Tam A.C., Richardson C.G. (2018). Gender, stressful life events and interactions with sleep: A systematic review of determinants of adiposity in young people. BMJ Open.

[57] Evans T.M., Bira L., Gastelum J.B., Weiss L.T., Vanderford N.L. (2018). Evidence for a mental health crisis in graduate education. Nat. Biotechnol..

[58] Eaton N.R., Keyes K.M., Krueger R.F., Balsis S., Skodol A.E., Markon K.E., Grant B.F., Hasin D.S. (2012). An invariant dimensional liability model of gender differences in mental disorder prevalence: Evidence from a national sample. J. Abnorm. Psychol..

[59] Woolston C. (2019). A better future for graduate-student mental health. Nature.

[60] Cao W., Fang Z., Hou G., Han M., Xu X., Dong J., Zheng J. (2020). The psychological impact of the COVID-19 epidemic on college students in China. Psychiatry Res..

[61] Swendsen J., Conway K.P., Degenhardt L., Glantz M., Jin R., Merikangas K.R., Sampson N., Kessler R.C. (2010). Mental disorders as risk factors for substance use, abuse and dependence: Results from the 10-year follow-up of the National Comorbidity Survey. Addiction.

[62] Stubbs B., Veronese N., Vancampfort D., Prina A.M., Lin P.Y., Tseng P.T., Evangelou E., Solmi M., Kohler C., Carvalho A.F. (2017). Perceived stress and smoking across 41 countries: A global perspective across Europe, Africa, Asia and the Americas. Sci. Rep..

[63] Choi D., Ota S., Watanuki S. (2015). Does cigarette smoking relieve stress? Evidence from the event-related potential (ERP). Int. J. Psychophysiol..

[64] Awaworyi Churchill S., Farrell L. (2017). Alcohol and depression: Evidence from the 2014 health survey for England. Drug Alcohol Depend..

[65] Gasmi A., Noor S., Tippairote T., Dadar M., Menzel A., Bjørklund G. (2020). Individual risk management strategy and potential therapeutic options for the COVID-19 pandemic. Clin. Immunol..

[66] Vardavas C.I., Nikitara K. (2020). COVID-19 and smoking: A systematic review of the evidence. Tob. Induc. Dis..

[67] Diviani N., van den Putte B., Giani S., van Weert J.C. (2015). Low Health Literacy and Evaluation of Online Health Information: A Systematic Review of the Literature. J. Med. Internet Res..

[68] Norman C.D., Skinner H.A. (2006). eHealth Literacy: Essential Skills for Consumer Health in a Networked World. J. Med. Internet Res..

[69] Duong T.V., Lin I.-F., Sørensen K., Pelikan J.M., Van den Broucke S., Lin Y.-C., Chang P.W. (2015). Health literacy in Taiwan: A population-based study. Asia-Pac. J. Public Health.

[70] Kayupova G., Turdaliyeva B., Tulerayev K., Duong T.V., Chang P.W., Zagulova D. (2017). Health literacy among visitors of district polyclinics in Almaty, Kazakhstan. Iran. J. Public Health.

[71] Watson R. (2011). Europeans with poor "health literacy" are heavy users of health services. BMJ.

[72] Greenhalgh T. (2015). Health literacy: Towards system level solutions. BMJ.

[73] Paakkari L., Okan O. (2020). COVID-19: Health literacy is an underestimated problem. Lancet Public Health.

[74] Sentell T., Vamos S., Okan O. (2020). Interdisciplinary Perspectives on Health Literacy Research Around the World: More Important Than Ever in a Time of COVID-19. Int. J. Environ. Res. Public Health.

[75] Wang C., Pan R., Wan X., Tan Y., Xu L., Ho C.S., Ho R.C. (2020). Immediate Psychological Responses and Associated Factors during the Initial Stage of the 2019 Coronavirus Disease (COVID-19) Epidemic among the General Population in China. Int. J. Environ. Res. Public Health.

