# Supplementary material for: Fear of COVID-19 Scale—Associations of Its Scores with Health Literacy and Health-Related Behaviors among Medical Students
Source: Int J Environ Res Public Health. 2020 Jun 11;17(11):4164. doi: 10.3390/ijerph17114164 (PMC7311979; doi:10.3390/ijerph17114164)
Supplement: Supplementary file 1 [file ijerph-17-04164-s001.pdf]

Table S1. Spearman correlations among covariates (N=5423).

	Age	Gender	Ability to pay	BMI	Academic year	S-COVID-19-S
Gender	-0.04					
Ability to pay	0.00	-0.01				
BMI	0.02	0.28	0.04			
Academic year	0.84	-0.06	0.02	0.01		
S-COVID-19-S	-0.04	-0.02	-0.03	-0.02	-0.05	
Comorbidity	0.00	0.03	0.01	0.01	0.00	0.09

Abbreviations: BMI, body mass index; S-COVID-19-S, suspected coronavirus disease-2019 symptoms
